# Why Do I Seek Negative Feedback? Assessment Orientation, Self-Criticism, and Negative Feedback-Seeking

**DOI:** 10.3389/fpsyg.2021.709261

**Published:** 2021-10-20

**Authors:** Zhaoyan Liu, Qinghong Yuan, Shanshan Qian, Molly Ellenberg, Arie W. Kruglanski

**Affiliations:** ^1^Business School, Nankai University, Tianjin, China; ^2^School of Business, Guangdong University of Foreign Studies, Guangzhou, China; ^3^Department of Psychology, University of Maryland, College Park, College Park, MD, United States

**Keywords:** negative feedback-seeking, assessment orientation, self-criticism, participative leadership, regulatory mode theory

## Abstract

Negative feedback plays an important role in employee performance improvement, yet little research has specifically examined the motivational factor that drives employees to seek negative feedback. Drawing from the regulatory mode theory, we propose that assessment orientation could increase negative feedback-seeking by triggering individual self-criticism and participative leadership could enhance this effect. Results from a two-wave lagged survey study obtained from 216 Chinese employees suggested that assessment orientation is positively correlated with negative feedback-seeking via the mediating role of self-criticism. Moreover, the positive effect of assessment orientation on self-criticism and the positive indirect effect of assessment orientation on negative feedback-seeking via self-criticism are both stronger when participative leadership is higher. These results enrich the literature on feedback-seeking and regulatory mode and are useful for increasing employee negative feedback-seeking behavior in the organization.

## Introduction

As organizations are faced with a dynamic and uncertain environment, specifically resulting from the outbreak and continuous spread of Coronavirus disease 2019, it is of vital importance to encourage employees’ proactive behavior. Feedback seeking is a kind of proactive behavior that has been recommended to improve employees’ job performance (Ashford, 1986; Ashford and Tsui, 1991; Lam et al., 2017; Ashford et al., 2018). Ashford and Tsui (1991) point that the feedback information that employees seek can be negative or positive according to its nature. Negative feedback-seeking refers to “employee’s report of his or her information seeking regarding areas that he or she underperforms” (Gong et al., 2017, p. 1235). Negative feedback has the functions of diagnosis and development (Ashford and Tsui, 1991; Moss et al., 2003), which can help employees understand their inadequacies in organizational tasks and adjust their works accordingly (Ashford et al., 2003). Existing studies have shown that negative feedback can increase job performance, leader effectiveness, and recipient creativity (Gong et al., 2017; Chun et al., 2018; Kim and Kim, 2020). Gong et al. (2017) also called for research about employee feedback-seeking pay more attention to the nature of feedback (negative or positive), which help us to understand feedback-seeking behavior more clearly and reduce the blurs of research results of the relationship between feedback-seeking and its related factors.

However, literature that paid attention to negative feedback-seeking is very small. Existing research on feedback-seeking has exclusively focused on frequency, approach, and source of feedback-seeking (e.g., Ashford et al., 2003, 2016; Anseel et al., 2015). Moreover, although a few exceptions explored the antecedents of negative feedback-seeking from a relational perspective, such as high-quality leader–member (Chen et al., 2007; Chun et al., 2018), it is still unclear whether motivational-related factors spark employees to seek negative feedback. Therefore, the purpose of this study is to explore whether, why, and when assessment orientation, a motivational factor from the perspective of individual self-regulation, influences employees’ negative feedback-seeking behavior.

Feedback-seeking in nature is an individual’s self-regulation process that involves evaluating themselves based on others’ information (Anseel et al., 2007; Ashford et al., 2016). Accordingly, regulatory mode theory, describing individual preference to adopt evaluation strategy in the process of regulation (Kruglanski et al., 2000, 2009), may offer an explanatory perspective for the motivational factors of employees seeking negative feedback. Regulatory mode theory argues that individuals have two orthogonal motivations in which they carry out the self-regulatory process: assessment orientation and locomotion orientation (Kruglanski et al., 2000; Higgins et al., 2003). Whereas locomotion orientation relates to movement between states, assessment orientation reflects the comparative function of self-regulation, that is, individuals evaluate the current situation by comparing alternatives to judge their relative quality, pursuing the truth and “the right thing to do” (Kruglanski et al., 2000; Higgins et al., 2003). Driven by assessment orientation, individuals will make behaviors conducive to evaluation and “to do the right thing” (Kruglanski et al., 2000, 2013; Scholl et al., 2021), for example, employees seek negative feedback about their adverse performance in the organization. Thus, we expect that assessment orientation acts as a positive predictor of negative feedback-seeking.

The present study examines how employees’ assessment orientation impacts negative feedback-seeking. Based on regulatory mode theory, we proposed that assessment orientation, emphasizing critical evaluation and comparison, increases employees’ negative feedback-seeking behavior because assessment orientation can trigger high levels of self-criticism. Furthermore, drawing from regulatory fit theory which emphasizing the fit between individual regulatory orientation and the manner to pursue a goal exerts a positive effect on the individual behavior, we proposed that participative leadership moderates the relationship between assessment orientation and its outcome. High levels of participative leadership provide a supportive environment that prompts employees to translate their motivation into related psychological activity and behaviors, making employees with the same level of assessment orientation undergo stronger self-criticism and thus seeking more negative feedback.

**FIGURE 1:**
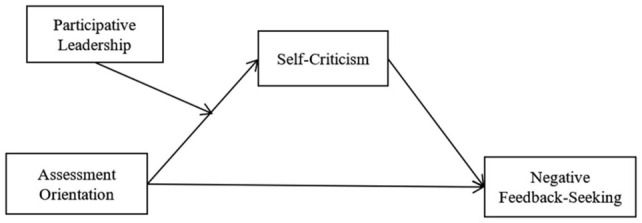
Hypothesized model.

This study makes contributions to the existing theory in the following areas. First, the present study extends the feedback-seeking literature by shifting the focus from the frequency, source, and extent of feedback-seeking to its nature. Specially, we explore how negative feedback-seeking occurs, identifying and examining assessment orientation as a motivational factor for negative feedback-seeking. Second, we reveal the underlying psychological mechanism about why assessment orientation motivates employees to seek negative feedback. Assessment orientation would spark employees’ self-criticism, which stimulates employees to solicit more negative feedback accordingly. Third, the findings identify participative leadership as the boundary condition, providing a more nuanced picture of how assessment orientation relates to employee negative feedback-seeking. Fourth, we advance regulatory mode theory by bridging it with feedback-seeking research for the first time. We provide empirical supports for the application of regulatory mode theory in the aspect of human resource management and add to the outcomes of assessment orientation. Figure 1 depicts our overall research model.

## Theory and Hypotheses Development

### Assessment Orientation and Negative Feedback-Seeking

Regulatory mode theory proposes that individuals have two independent regulatory modes in the process of achieving goals: assessment orientation and locomotion orientation (Kruglanski et al., 2000; Higgins et al., 2003). Assessment orientation is responsible for evaluation and comparison in the process of individual self-regulation (Carver and Scheier, 1990; Kruglanski et al., 2000). Regulatory mode theory also describes sociopsychological characteristics of assessment orientation. In terms of self-evaluation, assessors generally concentrate on repeatedly evaluating their actual selves by comparing themselves with other standards (e.g., expected self, colleagues in the same working group) (Higgins, 1987; Kruglanski et al., 2000). Furthermore, people with high assessment orientation emphasize the gap between the actual self and the desired self (Duval and Wicklund, 1972; Higgins, 1987). As a result, individuals with strong assessment orientation will experience obvious negative affect and lower self-esteem (Kruglanski et al., 2000, 2009; Pierro et al., 2018).

Although most of the current literature on assessment orientation and locomotion orientation has been concentrated in the field of social psychology (e.g., Pierro et al., 2011; Mathmann et al., 2017; Webb et al., 2017), an accumulating body of studies begins to apply it the organizational context (Kruglanski et al., 2007b; Chernikova et al., 2016; Lo Destro et al., 2016). However, the potential impact of regulatory mode on employees’ negative feedback-seeking has not yet been examined.

Based on regulatory mode theory, the present study posits that employees with a high score in assessment orientation tend to seek negative feedback for two reasons. First, because assessment orientation entails attempting to make the right choices through evaluation and comparison (Kruglanski et al., 2000; Higgins et al., 2003), assessors aim to correct inadequacies and demonstrate acceptable behavior in terms of role fulfillment and work performance. For example, individuals with assessment orientation were found to better detect the divergence between the self and the social norm when participating in gym classes (Kruglanski et al., 2009).

In an organization, the most common and effective way for employees to understand what they do not do well is to seek negative feedback from leaders and colleagues (Ashford and Cummings, 1985). Seeking negative feedback presents more attractive value for employees who want to improve their performance (Moss et al., 2003). The instrumental perspective in the feedback research argues that feedback is a great resource for employees to improve their work (Ashford and Cummings, 1983; VandeWalle et al., 2000; Ashford et al., 2003; Sung and Choi, 2021), and thus they are willing to take the initiative to receive feedback. Because employees high in assessment orientation aim to correct poor performance, these individuals should solicit more information about their inadequacies in the organization than individuals low in assessment orientation.

Second, employees with assessment orientation tend to compare themselves to high standards such as their expected self, the organizational requirements of employees, and colleagues who perform well, and take notice of the gap between the actual self and the ideal self (Higgins, 1987; Kruglanski et al., 2000). Consequently, individuals with high assessment orientation are more cognizant of what they did not do well. Furthermore, because they evaluate themselves repeatedly and compare themselves to their desired selves, individuals with high assessment orientation exhibit a negative self-view (Kruglanski et al., 2000, 2009). Existing empirical research has demonstrated that people high in assessment scores have stronger social anxiety and depression, and have lower levels of self-esteem and optimism (Kruglanski et al., 2009; Komissarouk et al., 2019; Santo et al., 2021). The principle of self-verification argues that people have a stable self-view and will strive to maintain their self-view to hold their sense of coherence. Swann et al. (1992) indicated that to verify themselves, people with negative self-views are more inclined to solicit unfavorable information about themselves, even if the negative information they receive will make them feel unpleasant. Therefore, we believe that employees high in assessment orientation will seek more negative information about themselves to verify their negative self-view, in addition to seeking to improve their performance based on evaluation and comparison. Pettit and Joiner (2001) also support this conclusion, suggesting that individuals experiencing a decline of self-esteem take the initiative to get unfavorable comments relating to their ability in social domains. In summary, we hypothesized the following:

**Hypothesis 1:** Assessment orientation will be positively associated with negative feedback-seeking.

### The Mediating Role of Self-Criticism

The present study also posits that self-criticism mediates the relationship between assessment orientation and negative feedback-seeking. Self-criticism refers to “the sensitivity to negative self-relevant information about where one has fallen short or failed to meet the standard of excellence shared in a given social unit” (Kitayama et al., 1997, p. 1246). It describes the personality characteristics that excessive concerns about autonomous achievement (Blatt et al., 1976; Blatt and Zuroff, 1992; Robins et al., 1994) and is influenced by higher-order personality traits, such as regulatory mode, reflecting the level of self-regulatory systems within a general personality architecture (Kruglanski et al., 2009, p. 398). Individuals high in self-criticism are acutely aware of behaviors that do not meet their own or others’ standards and tend to have a negative cognitive evaluation of themselves (Blatt and Zuroff, 1992; Blatt, 2004; Krieger et al., 2019; Löw et al., 2020).

According to regulatory mode theory, employees who have high scores in assessment orientation focus on critically evaluating themselves and are sensitive to their shortcomings, employees with high assessment orientation tend to be more critical of themselves than those low in assessment orientation (Kruglanski et al., 2000). Consistent with this view, self-assessment is considered a reflexive psychological behavior that can enhance individuals’ psychological tendency to criticize themselves, contributing to achieving desired goals (Lueke and Skeel, 2017). Moreover, Komissarouk et al. (2019) found that people with high assessment orientation and who aspire to do what is right exhibit high levels of self-criticism and low levels of self-esteem.

Self-verification literature contends that individual has a very powerful motivation to confirm their evaluation and cognition about themselves and they tend to seek evidence to prove rather than disprove their view (Swann and Read, 1981; Swann, 2011). An important way that people confirm their self-view is to seek social feedback from others, and feedback would be viewed as more valuable and compelling when it is consistent with their views (Swann and Read, 1981; Swann et al., 2003). Therefore, people with strong self-criticism are more likely to have a negative self-view, which motivates them to seek negative information about themselves from their leaders and colleagues to confirm their self-view. Prior studies provide indirect support for these propositions. Valentiner et al. (2011) suggest that university students with low social self-esteem have a higher preference for negative feedback than those students with high social self-esteem.

Furthermore, individuals high on self-criticism have a strong need for achievement and are devoted to constantly scrutinizing themselves (Mongrain and Zuroff, 1995). An achievement orientation that self-criticism inspires makes individuals focus on their faults. They are especially sensitive to the things they do not do well (Shahar et al., 2003). Lueke and Skeel (2017) reported that for people with a high level of self-criticism, feedback on a failure task caused an improvement of the following performance, and feedback on a success task resulted in a worsening of the following performance. A possible reason for this is that negative feedback meets these individuals’ needs for achievement and self-improvement. Likewise, Gong et al. (2017) demonstrated that employees who focus on criticizing their shortcomings seek more negative information from others because of the value of negative feedback in improving performance. Thus, the higher the level of self-criticism is, the more people prefer negative feedback about their performance.

Consistent with the above discussion, we expected that employees with an assessment orientation would tend to have a high level of self-criticism, which would directly encourage them to seek more negative feedback. Accordingly, we propose the following hypothesis:

**Hypothesis 2:** Self-criticism will mediate the positive relationship between assessment orientation and negative feedback-seeking.

### The Moderating Role of Participative Leadership

The regulatory fit theory contends that the fit between individuals’ regulatory orientation and the manner of goal pursuit makes individuals feel right about what they are doing and enhances their goal commitment behaviors (Higgins, 2005; Avnet and Higgins, 2006; Cesario et al., 2008). For example, Shin et al. (2017) found that when the employees’ regulatory focus, a motivational orientation, fits with that of their leaders’ regulatory focus, they maintain greater levels of organizational citizenship behavior.

In the context of organizational work, leaders are responsible for the performance evaluation of their subordinates and can determine how subordinates achieve their work goals to some extent (Morgeson et al., 2010; Hernandez et al., 2011; Beenen et al., 2017). When the leader encourages employees to achieve work goals in a way that matches employees’ regulatory orientation, the regulatory fit will achieve for employees. And thus, they will be satisfied with what they are doing, feel it is right, and enhance the strength of engagement in the goal-pursing activity. For example, Benjamin and Flynn (2006) showed that because the subordinates’ locomotion orientation fits with the leader’s transformational leadership style, the effectiveness of transformational leadership is highly evaluated by subordinates.

In Hypothesis 2, we figured that the individuals with strong assessment orientation are inclined to hold high self-criticism, leading them to seek more negative feedback about their work. According to the above discussion on regulatory fit theory and leadership, we believe leadership will moderate the relationship between assessment orientation and self-criticism, and the leadership that fits employees’ assessment orientation will enhance the effect of assessment orientation on self-criticism.

Participative leadership refers to leaders sharing the responsibility of solving problems and equalizing their power with subordinates by consulting with them to make decisions jointly (House, 1996; Sauer, 2011; Newman et al., 2016). A participative leader encourages subordinates to actively participate in problem-solving and gives subordinates extra attention and support, instead of giving them direct instructions about how to accomplish a task (Kahai et al., 2004; Lam et al., 2015; Buengeler et al., 2016; Lythreatis et al., 2019). In terms of employee performance evaluation, a participative leader will encourage employees to actively evaluate their work performance and fully discuss their performance with employees (Huang et al., 2010). According to regulatory mode theory, people with assessment orientation tend to critically evaluate entities or states included in goal-directed action (Kruglanski et al., 2000; Higgins et al., 2003). Thus, employees high in assessment orientation prefer participative leadership which allows employees to fully evaluate themselves, and provides support and discretion for employees.

According to regulatory fit theory, when participative leadership is high, employees with assessment orientation will achieve regulatory fit. The regulatory fit makes employees feel right about critically evaluating themselves and increased the strength to criticize themselves. That is, for employees with the same level of assessment orientation, a high (vs. low) participative leader provides a supportive environment, prompting them to translate their motivation of assessment orientation into stronger psychological activities of self-criticism. Therefore, when participative leadership is high, the relationship between assessment orientation and self-criticism will be enhanced.

On the contrary, when participative leadership is low, the leader can’t present s a favorable atmosphere for employees to fully evaluate themselves, which inhibits employees from translating their motivation of assessment orientation into the psychological activity of self-criticism. That is, when participative leadership is low, the relationship between assessment orientation and self-criticism will be weakened. The study by Kruglanski et al. (2007a) provides support for this by demonstrating that assessment orientation and participative leadership display significant interaction in predicting employees’ job satisfaction. Specifically, employees with assessment orientation have higher job satisfaction when their supervisors adopt the high (vs. low) participative leadership style because participative leaders allow assessors to evaluate themselves in the course of their work.

**Hypothesis 3**: Participative leadership will moderate the relationship between assessment orientation and self-criticism. This relationship will be stronger when participative leadership is high compared to when it is low.

As described by the moderating effect of participative leadership in hypothesis 3, when the level of participative leadership is high (vs. low), assessment orientation triggers employees’ stronger self-criticism. And, as described by the mediating effect of self-criticism in hypothesis 2, assessment orientation has an indirect effect on negative feedback-seeking via the role of self-criticism. That is, assessment orientation has a positive effect on self-criticism, and self-criticism has a positive effect on negative feedback-seeking. Therefore, when the level of participative leadership is high (vs. low), assessment orientation can lead to stronger self-criticism, which in turn drives employees to engage in more negative feedback-seeking behaviors. In other words, when led by the high (vs. low) participative leader, employees with the same level of assessment orientation seek more negative feedback by stronger self-criticism. The mediating role of self-criticism on the relationship between assessment orientation and negative feedback-seeking is enhanced by participative leadership. When the level of participative leadership is high, the mediating effect of self-criticism will be stronger. We build our hypothesis 4:

**Hypothesis 4**: Participative leadership will moderate the mediated relationship between assessment orientation and negative feedback-seeking through self-criticism such that the mediated relationship will be stronger under high levels of participative leadership than under low levels of participative leadership.

## Materials and Methods

### Samples and Procedures

We used the snowball sampling approach to recruit participants, enabling the researcher to obtain more heterogeneous data and improving the external validity of the study results (Lin et al., 2021). We contacted alumni of three universities in China who have worked through Wechat which is a very popular instant social software in China and asked them to recommend participants. Upon completion of that explanation of the purpose and procedures of our survey, 270 employees had agreed to participate in our research. We put measure items in electronic questionnaires and set up some rules, including identifying the IP address so that each participant can only answer once, and each item must be answered before submitting the questionnaire. Then we sent the website address of electronic questionnaires through WeChat. To reduce the Common Method Variance, we distributed the questionnaire at two points in time. At time 1, we sent questionnaires measuring demographic information, assessment orientation, and the participative leadership of their supervisor to all participants. After collecting the questionnaires, those questionnaires with too short response time, random responses, and wrong responses to the screening question were eliminated. As a result, 264 valid questionnaires were returned at time 1. Two weeks later at time 2, we sent the second round of questionnaires measuring self-criticism and negative feedback-seeking to the same 264 participants. After screening the second completed questionnaires and matching them with the first questionnaire through their employee IDs, 216 valid questionnaires were returned, yielding a total response rate of 80.00%.

Of the 216 respondents, the average age was 29.67 (*SD* = 4.31), the average organizational tenure in their current company was 4.00 years (*SD* = 3.64) 0.135 were men (62.5%) and 118 are married (54.6%). As for the educational background, 6 respondents had a high school degree, 39 respondents had a college diploma, 83 respondents had a bachelor’s degree, 78 respondents had a master’s degree and 10 respondents had a doctoral degree. These participants were from 39 cities in 20 provinces or municipalities in China. They were distributed across different industries, including hotel and catering services (24.50%), real estate (17.60%), public service (19.40%), energy production and supply (10.60%), manufacturing (6.90%), and those categorized as “others” (21.00%), and they were from different job positions, including the network operating and maintenance (24.50%), research and development (18.10%), decoration design (13.90%), education (10.60%), human resource management (6.90%), product production (5.60%), management (5.10%), and those categorized as “others” (15.30%).

### Measures

The measurements of assessment orientation, self-criticism, and participative leadership were the validated English versions of the scales. We conducted Brislin’s (1980) back-translation procedure to translate the English version of the scale into the Chinese version. Two graduate students majoring in English who were blind to our study completed the translation process. To begin with, one student translated the English version of the assessment orientation scale, self-criticism scale, and participative leadership scale into the Chinese version. Then, the other student translated Chinese versions of these scales into the English version. Finally, a Professor of Management with excellent command of English compared the two English versions, and finally confirmed the final Chinese version with only small modifications. This procedure has been widely used in prior studies (e.g., Tang et al., 2020). Negative feedback-seeking was measured with validated Chinese versions of the scale.

In the present study, all major study variables, excluding demographic variables, were measured using a five-point Likert type scale (1 = *strongly disagree*, 5 = *strongly agree*).

#### Assessment Orientation

Assessment orientation was assessed with the assessment subscale from the Regulatory Mode Questionnaire (Kruglanski et al., 2000). The previous study has demonstrated good reliability and validity in previous research in the Chinese context (Cronbach’s alpha was 0.77) (Li et al., 2018a). We used 11 items with high loading to measured assessment orientation. Sample item includes “I spend a great deal of time taking inventory of my positive and negative characteristics.” The Cronbach’s alpha for the assessment orientation scale was 0.71 in this study.

#### Self-Criticism

Self-criticism was assessed with the four-item self-criticism subscale from the Personal Style Inventory-II (PSI-II; Robins et al., 1994). The study from Cantazaro and Wei (2010) has demonstrated that adequate validity and reliability (Cronbach’s alpha was 0.76). Sample item of self-criticism includes “I have a hard time forgiving myself when I feel I haven’t worked up to my potential.” The Cronbach’s alpha for the self-criticism scale in this study was 0.78.

#### Participative Leadership

We asked employees to rate their leaders’ participative leadership using four items adapted from Oldham and Cummings (1996) and Kahai et al. (2004). Previous studies have shown good reliability and validity in the Chinese context (Cronbach’s alpha was 0.81) (Li et al., 2018a). Sample item includes “the team leader often allows our group members to have as much input into the final recommendation as he did.” The Cronbach’s alpha for the participative leadership scale in this study was 0.70.

#### Negative Feedback-Seeking

Negative feedback-seeking was rated using the six items used by Gong et al. (2017). Gong et al. (2017) developed the negative feedback-seeking scale with part-time MBA students and masters as subjects in the Chinese context and published the articles in the *Journal of Management*. They conducted two studies with employees and management as subjects in Chinese companies and conducted surveys in Chinese. The result of their study suggested that the scale of negative feedback-seeking has satisfying reliability (Cronbach’s alpha was 0.89). We received the Chinese vision of the negative feedback-seeking scale by sending an email to the corresponding author. The sample item is “I often indirectly ask for information on what I failed to perform.” The Cronbach’s alpha for the negative feedback-seeking scale in this study was 0.86.

#### Control Variables

Previous studies have shown that with the increase of age and organizational tenure of employees, they have stronger role clarity and perceive less value from feedback. As a result, they solicit less information about their work from others (Anseel et al., 2015). Thus, we take age and organizational tenure as control variables in the process of data analyses. As individuals with high education tend to seek less negative feedback than those with low education (Chen et al., 2007), we also controlled for the effect of education.

## Results

### Confirmatory Factor Analyses

To ensure eligible discriminant validity of the major study variables, confirmatory factor analysis was run firstly with the software Mplus 8.0. Item parceling makes the parameters be estimated more effectively for a small sample size (Little et al., 2002; Schmitt et al., 2016). Two unidimensional and long scales in the present study were parceled using the item-to-construct balanced approach which is recommended by Little et al. (2002) and widely used in previous studies (e.g., Li et al., 2018b; Qian et al., 2019). Specifically, 11 items of the assessment orientation scale and 6 items of the negative feedback-seeking scale were combined into three parcels respectively, and a total of 6 parcels were generated. Overall, the six parcels, four items of self-criticism scale, and four items of participative leadership scale were included in the confirmatory factor analysis. We examined a four-factor model and six three-factor models by combining any two of the four factors into one factor. As shown in Table 1, Results showed that the four-factor model fit the data well: χ*^2^*(71) = 119.87, *p* = 0.00 < 0.001, Comparative Fit Index = 0.95, Tucker–Lewis Index = 0.93, Root Mean Square Error of Approximation = 0.06 (90% CI [0.04,0.07]) and all indices were above the conventional cut-off values. Besides, the index of four-factor model is significantly better than alternative six three-factor models (110.87 ≤ Δχ*2* [Δ*df* = 3] ≤ 160.45, *p* < 0.001). These results suggested the major study variables had sufficient discriminate validity.

**TABLE 1:** Confirmatory factor analysis for discriminant validity.

**Model**	**χ*^2^*(*df*)**	**CFI**	**TLI**	**RMSEA [90% CI]**	**SRMR**	**Δχ*^2^* (Δ*df*)^a^**
Four-factor model (AO, SC, PL, and NFS)	119.87(71)	0.95	0.93	0.06[0.04,0.07]	0.05	
Three-factor model (AO and SC were combined)	230.74(74)	0.84	0.80	0.10[0.09,0.11]	0.08	110.87(3) ***
Three-factor model (AO and NFS were combined)	268.62(74)	0.80	0.75	0.11[0.10,0.13]	0.10	148.87(3) ***
Three-factor model (AO and PL were combined)	272.90(74)	0.79	0.74	0.11[0.10,0.13]	0.10	153.03.(3) ***
Three-factor model (SC and PL were combined)	270.92(74)	0.79	0.75	0.11[0.10,0.13]	0.10	151.05(3) ***
Three-factor model (SC and NFS were combined)	280.32(74)	0.78	0.73	0.11[0.10,0.13]	0.09	160.45(3) ***
Three-factor model (PL and NFS were combined)	265.70(74)	0.80	0.75	0.11[0.10,0.12]	0.10	145.83(3) ***

*N = 216.*

*^a^The chi-square difference for each model reflects its deviation from the four-factor model.*

*AO, assessment orientation; SC, self-criticism; PL, participative leadership; NFS, negative feedback-seeking.*

***p < 0.001.

### Harman’s One Factor Test

Assessment orientation and self-criticism are psychological variables and thus it is appropriate for employees to report by themselves. For the measurement of feedback-seeking behavior, some studies used others-rating measures (e.g., Ashford et al., 2018; Qian et al., 2020), and some studies use self-rating measures (e.g., Dimotakis et al., 2017; Sherf and Morrison, 2020; Sherf et al., 2020). Considering that employees may seek negative feedback in ways that are not perceived by leaders or colleagues, we believe that self-report measures can better reflect the situation of employee negative feedback-seeking and thus we ask employees to rate their feedback-seeking behavior. We adopt an employee-rating measure for participative leadership of their superior because employees are the recipients of leadership exerted by the superior. The data in this study is from a single source, which may lead to common method bias. We carried out Harman’s one-factor test to estimate it. The result suggests that the variance of one factor accounts for 20.06% of the total variance. Williams et al. (1989) contend that the proportion of method variance in total variance is about 25%. Thus, the common method bias in this study is reasonable and normal.

### Descriptive Statistical Analyses

Table 2 presents the means, standard deviations, correlations, and reliability coefficients for all study variables. As shown and consistent with the previous studies (Anseel et al., 2015), negative feedback-seeking was negatively related to organizational tenure (*r* = –0.14, *p* < 0.05). Although negative feedback-seeking was not significantly correlated with education (*r* = 0.08, *p* > 0.05) and age (*r* = –0.13, *p* > 0.05), self-criticism was significantly correlated with education (*r* = –0.15, *p* < 0.05) and age (*r* = –0.14, *p* < 0.05), and therefore we controlled for their effect. Besides, assessment orientation was positively related to self-criticism (*r* = 0.30, *p* < 0.01) and negative feedback-seeking (*r* = 0.17, *p* < 0.01). Self-criticism was positively correlated with negative feedback-seeking (*r* = 0.43, *p* < 0.01) and participative leadership was positively correlated with negative feedback-seeking (*r* = 0.13, *p* < 0.05). These findings provide rudimentary support for our hypotheses.

**TABLE 2:** Means, standard deviations, correlations, and alphas of variables.

	***Mean***	***SD***	**1**	**2**	**3**	**4**	**5**	**6**	**7**	**8**
(1) Gender	1.37	0.49								
(2) Age	29.67	4.31	–0.01							
(3) Education	3.22	0.89	0.10	–0.10						
(4) Organizational tenure	4.00	3.65	–0.06	0.59*	−0.17*					
(5) Assessment orientation	2.92	0.50	0.04	−0.21**	0.05	–0.04	(0.71)			
(6) Self-criticism	3.11	0.75	0.06	−0.14*	−0.15*	−0.15*	0.30**	(0.78)		
(7) Participative leadership	3.52	0.63	–0.05	0.01	–0.04	–0.06	0.05	0.09	(0.70)	
(8) Negative feedback-seeking	3.54	0.69	0.03	–0.13	0.08	−0.14*	0.17**	0.43**	0.13*	(0.86)

*N = 216.*

*Gender was coded “1” for men and “2” for women. Education was coded “1” for “high school diploma or below,” “2” for “college diploma,” “3” for “bachelor degree,” “4” for “master degree,” “5” for “doctor degree.”*

*^a^Reliability coefficients are reported along the diagonal.*

*p < 0.05; **p < 0.01.

### Hypotheses Tests

To test Hypothesis 1, this study employed a hierarchical regression analysis using SPSS 20.0. As presented in Model 2 shown in Table 3, after controlling for age, education, and organizational tenure, assessment orientation was positively associated with negative feedback-seeking (β = 0.21, *SE* = 0.10, *p* < 0.05), supporting Hypothesis 1.

**TABLE 3:** The effect of assessment orientation on negative feedback-seeking.

**Predictors**	**Outcome: Negative feedback-seeking**	
	**Model 1**	**Model 2**
Constant	3.81 (0.41)***	3.02 (0.54)***
Age	–0.01 (0.01)	–0.01 (0.01)
Education	0.05 (0.05)	0.04 (0.05)
Organizational tenure	–0.02 (0.02)	–0.02 (0.02)
Assessment orientation		0.21 (0.10)*
*R* ^2^	0.03	0.05*
△*R*^2^	0.03	0.02*

*N = 216.*

*Unstandardized coefficients are presented; Standard errors are reported in parentheses.*

*p < 0.05; ***p < 0.001.

Hypothesis 2 predicted that self-criticism would mediate the relationship between assessment orientation and negative feedback-seeking. We used Model 4 in Hayes’ (2013) PROCESS macro to test it. As shown in Table 4, the indirect relationship between assessment orientation and negative feedback-seeking through self-criticism was 0.18 (*SE* = 0.05, 95% confidence interval [*CI*] [0.09–0.30]). Because the 95% *CI* did not contain 0, Hypothesis 2 was supported.

**TABLE 4:** The mediating effect of self-criticism.

**Predictors**	**Outcome: Self-criticism**	**Outcome: Negative feedback-seeking**
Constant	2.44 (0.55)***	2.05 (0.52)***
Age	–0.00 (0.01)	–0.01 (0.01)
Education	–0.16 (0.05)**	0.11 (0.05)*
Organizational tenure	–0.03 (0.02)	–0.01 (0.01)
Assessment Orientation	0.45 (0.10)***	0.03 (0.10)
Self-criticism		0.40 (0.06)***
*R* ^2^	0.13***	0.21***
Bootstrapping indirect effects	Boot SE	Boot 95% CI
0.18	0.05	[0.09,0.30]

*N = 216. Bootstrap N = 5,000.*

*Unstandardized coefficients are presented; Standard errors are reported in parentheses.*

*p < 0.05; **p < 0.01; ***p < 0.001.

**FIGURE 2:**
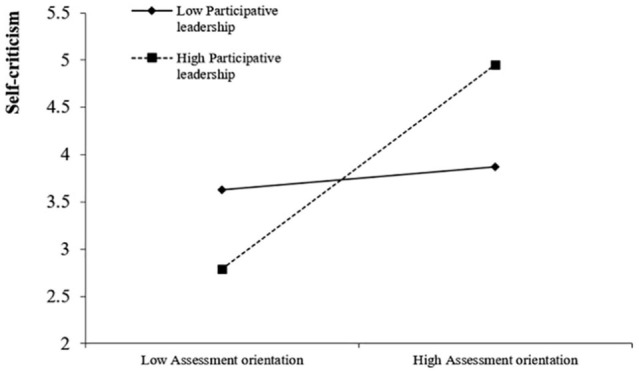
Interaction effect of assessment orientation and participative leadership on self-criticism.

We ran a hierarchical regression analysis with SPSS to test Hypothesis 3. To reduce the degree of multicollinearity of the variance inflation factor, independent variable, moderating variable, and interaction between them were centered in moderating effect analysis (Aiken et al., 1991; Robinson and Schumacker, 2009). Centering is defined as subtracting the mean (a constant) from each score, yielding a centered score (Robinson and Schumacker, 2009). Specifically, centered assessment orientation is equal to the original data of assessment orientation minus its mean, centered participative leadership is equal to the original data of participative leadership minus its mean, and the interaction term is equal to the product of the centered assessment orientation and the centered participative leadership. We set self-criticism as the dependent variable of the equation. In step 1, we put age, education, and organizational tenure in the regression equation. In step 2, we first put centered assessment orientation and centered participative leadership into the regression equation. In step 3, we put the interaction term of centered assessment orientation and centered participative leadership to the regression equation. As shown in Model 5 of Table 5, the interaction between assessment orientation and participative leadership had a significantly positive effect on self-criticism (β = 0.30, *SE* = 0.14, *p* < 0.05). To further assess the interaction effect, we conducted simple slope analyses (Aiken et al., 1991). As shown in Figure 2, the relationship between assessment orientation and self-criticism was not significant when participative leadership was low (–1 *SD*; β = 0.21, *SE* = 0.15, *p* > 0.05), whereas the relationship was significant when participative leadership was high (+1 *SD*; β = 0.60, *SE* = 0.12, *p* < 0.001). Therefore, Hypothesis 3 was supported.

**TABLE 5:** The moderating effect of participative leadership.

**Predictors**	**Outcome: Self-criticism**		
	**Model 3**	**Model 4**	**Model 5**
Constant	4.13 (0.44)***	3.77 (0.43)***	3.64 (0.43)***
Age	–0.02 (0.01)	–0.00 (0.01)	0.00 (0.01)
Education	–0.15 (0.06)*	–0.16 (0.06)**	–0.15 (0.05)**
Organizational tenure	–0.03 (0.02)	–0.03 (0.02)	–0.03 (0.02)
Assessment orientation		0.45 (0.10) ***	0.40 (0.10)***
Participative leadership		0.07 (0.08)	0.05 (0.08)
Assessment orientation × Participative leadership			0.30 (0.14)*
*R* ^2^	0.06**	0.14***	0.16***
△R^2^	0.06**	0.09***	0.02*

*N = 216.*

*Unstandardized coefficients are presented; Standard errors are reported in parentheses.*

*p < 0.05; **p < 0.01; ***p < 0.001.

We used Model 7 in Hayes’ (2013) PROCESS macro tested the indirect effect of assessment orientation on negative feedback-seeking through the mediating role of self-criticism at different levels of participative leadership. As shown in Table 6, at a low level of participative leadership, assessment orientation did not have a significant indirect effect on negative feedback-seeking through self-criticism (β = 0.08, *SE* = 0.06, 95% *CI* [–0.03 –0.22]). In contrast, at a high level of participative leadership, assessment orientation had a significant indirect effect on negative feedback-seeking through self-criticism (β = 0.24, *SE* = 0.06, 95% *CI* [0.12 –0.37]). The index of moderated mediation is 0.12 (*SE* = 0.05, 95% *CI* [0.02 –0.23]). Together, the results suggest that the indirect effect of assessment orientation on negative feedback-seeking through the mediating role of self-criticism will be stronger when participative leadership is high. These findings support Hypothesis 4.

**TABLE 6:** The indirect effect in different levels of participative leadership.

**Participative leadership**	**Conditional indirect effects**		
	**Indirect effects**	**Boot SE**	**Boot 95% CI**
Low (mean – 1 SD)	0.08	0.06	[–0.03,0.22]
Mean	0.16	0.05	[0.07,0.27]
High (mean + 1 SD)	0.24	0.06	[0.12,0.37]

*N = 216. Bootstrap N = 5,000.*

*Unstandardized coefficients are presented.*

## Discussion

Our study shifted the focus of research from the frequency, resource, and extent of feedback-seeking to negative feedback-seeking which emphasizes the nature of feedback and explored the motivation of negative feedback-seeking. Premised on regulatory mode theory (Kruglanski et al., 2000; Higgins et al., 2003), this study explored how assessment orientation affects employees’ negative feedback-seeking and the role of self-criticism and participative leadership in the relationship between assessment orientation and negative feedback-seeking. The results of our study showed that assessment orientation was positively related to employees’ negative feedback-seeking and self-criticism mediated the positive relationship between assessment orientation and negative feedback-seeking. Furthermore, participative leadership, as a moderator, enhanced the positive relationship between assessment orientation and negative feedback-seeking through self-criticism.

### Theoretical Implications

The present study makes theoretical contributions to the existing literature on feedback-seeking and regulatory mode. First, we add new knowledge to understand the full picture of employee feedback-seeking. Scholars have primarily paid attention to how to motivate employees to solicit feedback information more generally without sufficient attention to the nature (i.e., negative and positive) of feedback being sought (Anseel et al., 2015; Ashford et al., 2016). Gong et al. (2017) suggested that the exclusive focus on the nature of feedback is also important to fully understand the feedback-seeking behavior. In response to the call, we shift focus from the frequency, extent, and source to the nature of feedback-seeking, specifically paying close attention to negative feedback-seeking, and thus enhancing the understanding of the different aspects of feedback-seeking. In addition, the research that examines the effect of motivational factors on negative feedback-seeking has been rather limited so far. Drawing into regulatory mode theory (Kruglanski et al., 2000; Higgins et al., 2003), this study examines the relationship between assessment orientation and negative feedback-seeking and the empirical result suggests that assessment orientation positively predicts negative feedback-seeking. This finding provides one possible motivational factor for employees seek negative feedback.

Second, this study contributes to the psychological mechanism explaining why assessment orientation functions in predicting employees’ negative feedback-seeking. We find that employees high in assessment orientation are more likely to criticize themselves and high self-criticism drives employees to seek more negative feedback about their performance to ensure self-verification and a sense of achievement (Shahar et al., 2003; Swann, 2011).

Third, this study also brings new insights into boundary conditions regarding when assessment orientation can predict self-criticism and negative feedback-seeking. Drawing to regulatory fit theory (Higgins, 2005; Cesario et al., 2008), we identify an important leader factor- participative leadership could be an important moderator. When the level of participative leadership is high, assessment orientation significantly affected self-criticism and significantly affected negative feedback-seeking via self-criticism, but when the level of participative leadership is low, the effect of assessment orientation on self-criticism and the indirect effect of assessment orientation on negative feedback-seeking via self-criticism was not significant.

Fourth, we advance not only the literature about feedback-seeking but also the regulatory mode theory. While some studies have introduced regulatory mode (i.e., assessment orientation and locomotion orientation) into the field of organizational behavior (e.g., Lo Destro et al., 2016; Li et al., 2018a; Kanze et al., 2019), our study provides new evidence for the theory’s predictive value relating to employee negative feedback-seeking, thus expanding the application range of regulatory mode theory. Specifically, we found that assessment orientation, which emphasizes critical comparison and evaluation in the process of self-regulation, positively predicts negative feedback-seeking behavior in employees.

### Practical Implications

Our study also is helpful to the personnel recruitment, management, and self-management of employees. First, individuals differ in their level of assessment orientation, and this is a stable personality trait affected by general personality patterns (e.g., the Big Five personality factors) (Kruglanski et al., 2009). The results indicate that individuals with high assessment, inclined to engage in stronger self-criticism, are more willing to seek negative information about their performance. Thus, when organizations recruit employees for positions that require workers to constantly monitor work behavior and identify deficiencies (for example, stockbrokers, public traffic drivers, accountants, and auditors), we recommend that organizations evaluate the level of assessment orientation of candidates and give preference to candidates with a high assessment orientation.

Second, our study suggested that participative leadership which acts as a moderator can enhance the relationship between assessment orientation and its outcomes. This means that translating motivation into related work behaviors by employees in the organizational situation requires appropriate leadership styles. When leaders adopt highly participative leadership as a management strategy, employees with assessment orientation will experience stronger self-criticism and engage in more negative feedback-seeking, contributing to performance correction and improvement. Thus, leaders should deliver a more participatory management strategy for employees who prefer to use assessment orientation, which is beneficial to their positive work behavior.

Finally, our study also provides implications for job seekers. We believe that job seekers need understand which regulatory orientation they prefer to adopt, the assessment orientation, or the locomotion orientation. Individuals high in assessment orientation are good at evaluating themselves and soliciting negative information about their performance, and thus they are better suited for jobs that ask the worker to evaluate and rectify deficiencies promptly, such as jobs related financial duties, security duties. If job seekers know their regulatory mode well, it is more conducive to find jobs that give full play to their advantages.

### Limitations and Future Research

The present study has several limitations. First, although we measured variables at two time points, all data in this study was self-rated by employees. Data obtained from a single source may produce common method biases that have potentially negative effects on the analysis results (Podsakoff et al., 2003). As employees rated their work behavior, we inevitably worry about the influence of social desirability on the data, wherein the individuals respond inconsistently with their actual behavior to obtain social approval and present positive images of themselves (Crowne and Marlowe, 1964). For instance, the dependent variable in our study – negative feedback-seeking of employees – was scored by the employees themselves, and they may have over-reported their negative feedback-seeking behavior. Therefore, further research should aim to collect data from varied sources. For example, increasing numbers of studies have used leader–follower dyadic data (e.g., Eva et al., 2019), which can effectively minimize common method bias.

Second, instead of tracking and measuring the same concept at some time points, we measured different concepts at two time points to reduce the common method variance. Therefore, the research design is cross-sectional in nature and does not allow causal inferences about the proposed relationships between study variables. Regulatory mode theory posits that assessment orientation can also be evoked situationally (Avnet and Higgins, 2003) and prior research has effectively manipulated the level of assessment orientation (e.g., Webb et al., 2017). We encourage future studies to adopt both cross-sectional surveys and laboratory experiments to further enhance the reliability and causality of research results.

Third, we emphasized the importance of focusing on the nature of feedback-seeking but our study explored only negative feedback-seeking and neglected positive feedback-seeking, which limits the understanding of when and why people seek positive versus negative feedback. Simultaneously measuring negative and positive feedback-seeking could be one future research direction. Moreover, although negative feedback can help employees correct their inadequacies in work tasks, it has affective costs (Belding et al., 2015; Wakeling et al., 2020). For example, receiving negative feedback can increase individuals’ negative emotions and counterproductive work behaviors, and decreases individuals’ improvement self-efficacy (Belschak and Den Hartog, 2009; Dimotakis et al., 2017). It is noteworthy that seeking negative feedback and receiving negative feedback are different processes and it is not clear that whether seeking negative feedback can be detrimental, as receiving such feedback can be. Therefore, further research should explore the potential “double-edged sword” effect of seeking negative feedback.

Fourth, the participants in our study were all from China, so it is not clear whether our findings still hold in other cultural contexts. Cross-cultural research on regulatory orientation showed that Korean and Japanese have a high score in assessment orientation and low score in locomotion orientation, while Italian, Spanish, and Indian have a high score in locomotion orientation and low score in assessment orientation (Kruglanski et al., 2009). Cross-cultural studies about self-construction suggested that Easterners are more involved in self-criticism, while Westerners are more involved in self-enhancement (Kitayama et al., 1997; Heine et al., 1999; Heine, 2001). Therefore, it is not known whether the relationship between assessment orientation and self-criticism and negative feedback-seeking verified in this study is cross-culture. Future studies about regulatory orientation or self-criticism should investigate subjects in different cultural contexts to ensure the generalizability of our findings.

Fifth, we focused on the effect of assessment orientation on negative feedback-seeking but we didn’t control for the effect of locomotion orientation. Regulatory mode theory suggests that assessment orientation and locomotion orientation operate independently, and thus they may compete for some resource, such as time, attention, causing the inhibition of one to another (Kruglanski et al., 2009). Therefore, future research should also consider controlling the effect of locomotion orientation (assessment orientation) when only focusing on the main effect of assessment orientation (locomotion orientation).

Finally, the variance explained by the assessment orientation accounts for 2% of the total variance of negative feedback-seeking in Model 2. Although it reaches a statistically significant level (Δ*R^2^* = 0.02, *p* < 0.05), this percentage is still small, suggesting that there may be other key variables that can better predict employees’ negative feedback-seeking behavior. Similarly, the variance explained by the interaction term between assessment orientation and participative leadership accounts for 2% of the total variance of self-criticism (Δ*R^2^* = 0.02, *p* < 0.05), also suggesting that there may be other more critical moderating variables in the relationship between assessment orientation and self-criticism. Therefore, future research should explore more factors that influence employees’ negative feedback-seeking and more moderating variables influencing the relationship between assessment orientation and self-criticism or negative feedback-seeking from different perspectives.

## Data Availability Statement

The raw data supporting the conclusions of this article will be made available by the authors, without undue reservation.

## Ethics Statement

Ethical review and approval was not required for the study on human participants in accordance with the local legislation and institutional requirements. The patients/participants provided their written informed consent to participate in this study. Written informed consent was obtained from the individual(s) for the publication of any potentially identifiable images or data included in this article.

## Author Contributions

ZL, QY, SQ, and AK: research design. ZL: data collection. ZL and SQ: data analysis. ZL: writing of the original draft. ZL, QY, SQ, ME, and AK: revising article. All authors contributed to the article and approved the submitted version.

## Conflict of Interest

The authors declare that the research was conducted in the absence of any commercial or financial relationships that could be construed as a potential conflict of interest.

## Publisher’s Note

All claims expressed in this article are solely those of the authors and do not necessarily represent those of their affiliated organizations, or those of the publisher, the editors and the reviewers. Any product that may be evaluated in this article, or claim that may be made by its manufacturer, is not guaranteed or endorsed by the publisher.
